# Revealing the functional potential of microbial community of activated sludge for treating tuna processing wastewater through metagenomic analysis

**DOI:** 10.3389/fmicb.2024.1430199

**Published:** 2024-07-19

**Authors:** Zhangyi Zheng, Changyu Liao, Yubin Chen, Tinghong Ming, Lefei Jiao, Fei Kong, Xiurong Su, Jiajie Xu

**Affiliations:** ^1^School of Marine Science, Ningbo University, Ningbo, Zhejiang, China; ^2^Microbial Development and Metabolic Engineering Laboratory, Ningbo University, Ningbo, Zhejiang, China

**Keywords:** tuna processing, wastewater, activated sludge, metagenomics, nitrogen removal

## Abstract

Reports regarding the composition and functions of microorganisms in activated sludge from wastewater treatment plants for treating tuna processing wastewater remains scarce, with prevailing studies focusing on municipal and industrial wastewater. This study delves into the efficiency and biological dynamics of activated sludge from tuna processing wastewater, particularly under conditions of high lipid content, for pollutant removal. Through metagenomic analysis, we dissected the structure of microbial community, and its relevant biological functions as well as pathways of nitrogen and lipid metabolism in activated sludge. The findings revealed the presence of 19 phyla, 1,880 genera, and 7,974 species, with Proteobacteria emerging as the predominant phylum. The study assessed the relative abundance of the core microorganisms involved in nitrogen removal, including *Thauera* sp. MZ1T and *Alicycliphilus denitrificans* K601, among others. Moreover, the results also suggested that a diverse array of fatty acid-degrading microbes, such as *Thauera aminoaromatica* and *Cupriavidus necator* H16, could thrive under lipid-rich conditions. This research can provide some referable information for insights into optimizing the operations of wastewater treatment and identify some potential microbial agents for nitrogen and fatty acid degradation.

## 1 Introduction

Tuna processing wastewater is classified as non-toxic and biodegradable sewage with high dissolved organic matter, and it contains significant amounts of organic substances, including lipids, proteins, sugars, and amino acids, with lipids being the most abundant component (Fukada et al., 2016). The discharge of such wastewater is highly detrimental to agriculture and food industries, making it one of the most destructive forms of industrial wastewater (Sayana and Sirajudheen, 2017; Menon, 2021). The release of nitrogen and phosphorus has the potential to cause eutrophication of surface water and groundwater contamination, posing significant environmental concerns (Sirohi et al., 2022). Furthermore, the excessive discharge of lipids originating from tuna processing wastewater not only burdens water treatment facilities but also poses substantial risks to both the environment and human health (Donnarumma et al., 2021). Removing lipids is challenging due to their low density and poor solubility. In such cases, biological treatment, such as the activated sludge process, is a commonly used and effective method in wastewater treatment plants.

Activated sludge is widely used in the field of wastewater treatment, which harbors a diverse microbial community crucial for degrading pollutants, including organic matter, nitrogen, phosphorus, and sulfur, found in wastewater (Fan et al., 2020). Nitrogen removal microorganisms in the activated sludge facilitate the conversion of ammonia nitrogen and nitrite into nitrate or molecular nitrogen (Fan et al., 2020; Dai et al., 2022). Certain facultative microorganisms, as reported, possess lipase secretion capabilities, which aid in lipid degradation into fatty acids, and these fatty acids are subsequently broken down by specific functional microorganisms (Chipasa and Medrzycka, 2008; Guo et al., 2015). Additionally, the performance of activated sludge is contingent upon various functional microorganisms, significantly impacting the overall operational efficiency of wastewater treatment facilities (Dottorini et al., 2023). Analyzing the microbial structure and function of activated sludge can offer a robust assessment of wastewater treatment system performance.

Currently, numerous studies are dedicated to investigating sludge microorganisms, particularly focusing on nitrogen metabolism pathways (Dai et al., 2022). Research on the nitrogen metabolism pathway of activated sludge is essential for understanding wastewater treatment mechanisms (Wilén et al., 2018). Tian and coworkers identified 51 phyla and nearly 900 genera by using metagenomic analysis in a full-scale A2O sludge sample from the Lianyungang sewage treatment plant in Jiangsu province of China, and successfully predicted genes and strains related to nitrogen removal (Tian et al., 2015; Perez-Esteban et al., 2022). Additionally, previous research revealed the composition of activated sludge communities with temporal variations in a municipal sewage treatment facility over a 4-year period using metagenomic analysis (Ju et al., 2014). Furthermore, a recent study has investigated the effect of microplastics and nanoplastics on the microbial nitrogen conversion and metabolism of the activated sludge (Huang et al., 2023). However, existing research on the microbiological composition and operational characteristics of activated sludge primarily focuses on domestic, industrial, and municipal wastewater (Tian et al., 2015; Guo et al., 2017). Unfortunately, there is a lack of scientific investigations into sludge dynamics associated with the treatment of food processing wastewater. Additionally, research on typical lipid metabolism processes and related microorganisms is scarce.

It is widely acknowledged that metagenomics can allow for direct sequencing of the microorganisms present in the sample, thereby enabling a more comprehensive and extensive understanding of structure and functional capabilities of microbial communities (Akaçin et al., 2022; Liu et al., 2022; Lema et al., 2023). Therefore, this study employed metagenomic sequencing to assess the microbial community from activated sludge sample obtained from a wastewater treatment unit in a tuna processing plant in Ningbo, China. The microbial community structure, functional characteristics, and major metabolic pathways of activated sludge were comprehensively analyzed. Specifically, the study emphasized the analysis of functionally relevant microorganisms involved in nitrogen and lipid metabolism, laying a theoretical foundation for enhancing nitrogen and lipid removal efficiency and identifying highly efficient degradation microorganisms. The findings of this research provide valuable insights into the unique mechanisms by which activated sludge improves water quality in environments with high lipid content.

## 2 Materials and methods

### 2.1 Sampling of activated sludge

The activated sludge sample used in this experiment was collected from the site of the secondary clarifier, originating from a tuna processing wastewater treatment plant in Ningbo, China. The wastewater treatment plant employs the traditional anaerobic/aerobic (A/O) process with anaerobic and aerobic section tanks, as reported previously (Fan et al., 2020). The components of activated sludge were analyzed as shown in Supplementary Table S1, and the detection indicators of upper water at the sampling site was presented in Supplementary Table S2.

### 2.2 Metagenomics

#### 2.2.1 DNA extractions

DNA extraction from various samples was carried out using the E.Z.N.A.® Stool DNA Kit (D4015-02, Omega, Inc., USA) following the manufacturer’s instructions. The reagent in this kit, tailored for uncovering DNA in minute sample quantities, has been validated for DNA extraction across a wide range of bacterial species. Sample blanks, consisting of unused swabs, underwent DNA extraction and subsequent examination to confirm the absence of DNA amplicons. The elution of total DNA was performed using 50 μL of elution buffer following by a modified version of the protocol provided by the manufacturer (QIAGEN). Subsequently, the extracted DNA was stored at −80°C until PCR analysis, conducted by LC-Bio Technologies (Hangzhou) Co., Ltd., located in Hang Zhou, Zhejiang province, China.

#### 2.2.2 DNA library construction, sequencing, and assembling

The DNA library construction utilized the TruSeq Nano DNA LT Library Preparation Kit (FC-121-4001). Initially, DNA fragmentation was achieved using dsDNA Fragmentase (NEB, M0348S) with incubation at 37°C for 30 min. Blunt-ended DNA fragments were then generated through a combination of fill-in processes and exonuclease activity, followed by size selection using purification beads provided in the kit. Subsequently, A-base addition to the blunt ends facilitated ligation with indexed adapters. These adapters, featuring T-base overhangs, enabled ligation to the A-tailed fragmented DNA, incorporating sequencing primer hybridization sites for single, paired-end, and indexed reads. Fragments were ligated with single-or dual-index adapters, followed by PCR amplification of the ligated products. Sample sequencing was performed on an Illumina HiSeq 4000 platform (San Diego, CA, United States) by following the manufacturer’s recommendations, provided by LC-Bio. Raw sequencing reads underwent processing to obtain a set of valid reads for subsequent analysis. Sequencing adapters were initially removed using cutadapt v1.9 software, and low-quality reads were trimmed by fqtrim v0.94 using a sliding-window technique. Additionally, reads were aligned to the host genome using bowtie2 v2.2.0 to exclude potential host contamination. Quality-filtered reads were then used to construct the metagenome for each sample through *de novo* assembly using SPAdes v3.10.0.

#### 2.2.3 Taxonomic classification and gene function annotation

The prediction of all coding regions (CDS) of metagenomic contigs were performed using MetaGeneMark v3.26. CDS sequences of all samples were clustered by CD-HIT v4.6.1 to obtain unigenes. The estimation of unigenes abundance for a specific sample was conducted using the metric of the transcripts per million, which was calculated based on the number of aligned reads obtained using bowtie2 v2.2.0. The determination of the lowest common ancestor taxonomy of unigenes was achieved through the alignment of these sequences against the NCBI NR database using DIAMOND v0.7.12. The abundance of all genes was compared using the KO database, and the functional genes and key enzymes related to nitrogen and lipid metabolism were manually identified.

## 3 Results

### 3.1 Overview of metagenomic data and microbial composition in the activated sludge

After quality control filtering, a total of 69,336,498 raw data was obtained. Raw data was preprocessed to remove the joint sequences and low-quality data from sequencing reads, and a total of 63,351,450 clean data was obtained. The effective data rate was 91.37%, including 97.95% of data with effective data quality ≥ Q20 and 94.37% of data with effective data quality ≥ Q30. After assembling the sequencing data through IDBA-UD, the total contigs were up to 201,462 (Table 1). The N50 and N75 values were 1,196 bp and 713 bp, respectively. MetaGeneMark was used to predict the coding region of the sample. According to the predicted result, CD-HIT software was used to remove redundancy and unigene was obtained after identity 95% and coverage 90% were clustered. The number of open reading frames was 342,169 in unigene. The number of complete genes was 116,069, accounting for 33.92%. And the total length of genes and the average length of genes were 201.41 Mbps and 588.42 bps, respectively.

**Table 1:** The summary results of contig assembly and unigene catalog.

	Item	Number
Contig	Contig number	201,462
	N50 (bp)	1,196
	N75 (bp)	713
	Max Len. (bp)	168,619
Unigene	ORFs NO.	342,169
	Complete genes number	116,069 (33.92%)
	Total Len. (Mbp)	201.41
	Average Len. (bp)	588.42

To gain a deeper understanding of the community structure, taxonomic relationships were examined. A total of 19 phyla, 1,880 genera, and 7,974 species were identified. The microorganisms present in the activated sludge from tuna processing wastewater predominantly consisted of bacteria, viruses, archaea, and eukaryotes. Among these, bacteria exhibited the highest relative abundance, comprising 98.8% of the total microorganisms. Conversely, the proportions of viruses, archaea, and eukaryotes were relatively lower in the activated sludge, accounting for 0.8, 0.2, and 0.06%, respectively (Supplementary Figure S1).

### 3.2 Bacterial composition and abundance in the activated sludge

**Figure 1:**
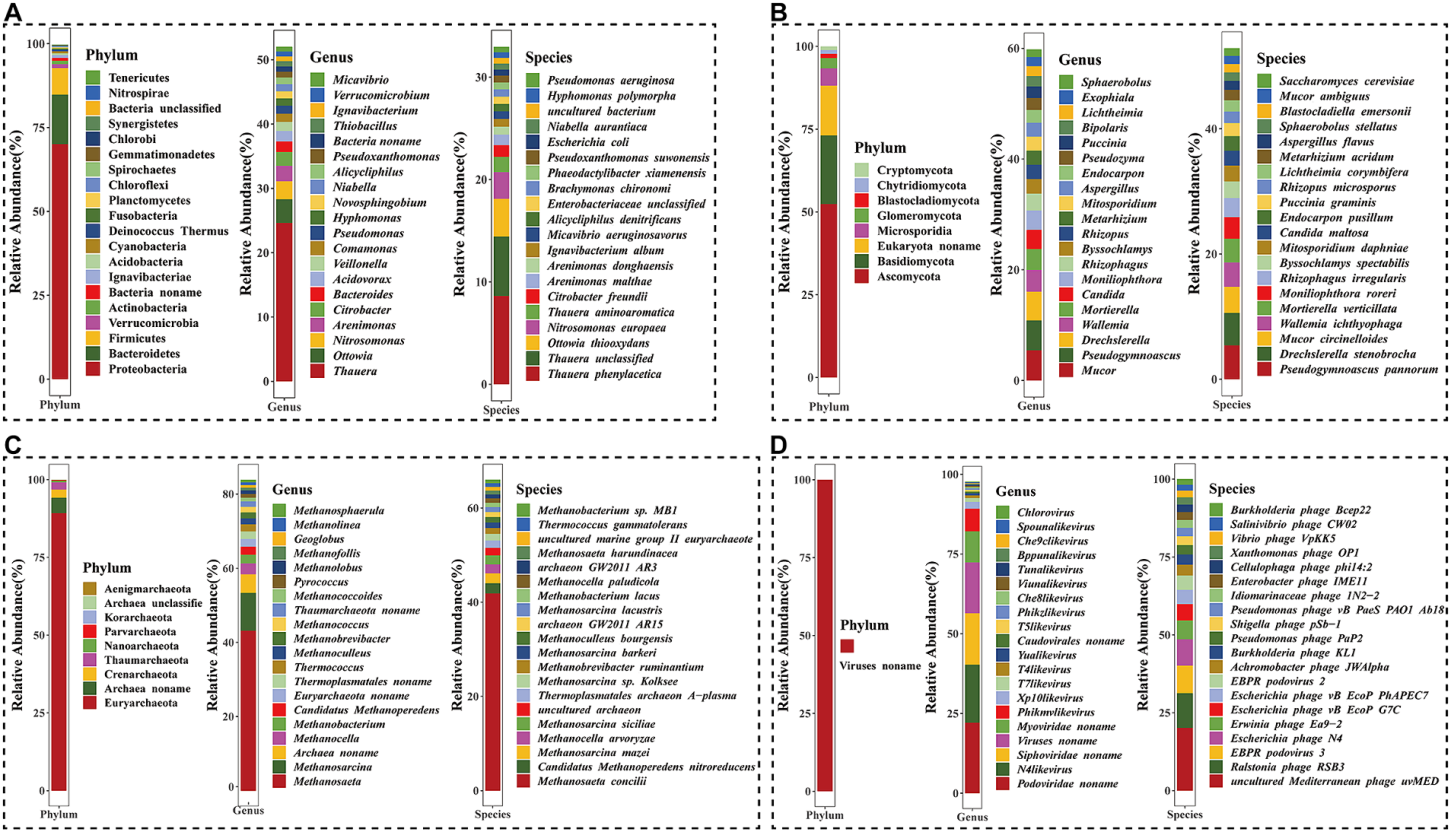
Microbial community composition of four kingdoms at the phylum, genus, and species levels. **(A)** Bacteria kingdom; **(B)** Eukaryota kingdom; **(C)** Archaea kingdom; **(D)** Viruses kingdom.

Bacteria accounted for the highest proportion in the activated sludge. At the bacterial phylum level, the predominant phyla included Proteobacteria, Bacteroidetes, Firmicutes, and Verrucomicrobia (Figure 1A). Proteobacteria was the most dominant phylum, representing 70.1% of the total, while the relative abundances of Bacteroidetes, Firmicutes, and Verrucomicrobia were 14.8, 7.9, and 1.2%, respectively.

At the genus level, a total of 1,600 different genera were identified (Figure 1A). Among these, dominant bacteria genera with a relative abundance of ≥1% included *Thauera* (24.9%), *Ottowia* (3.72%), *Nitrosomonas* (2.83%), *Arenimonas* (2.42%), *Citrobacter* (2.19%), *Bacteroides* (1.67%), *Acidovorax* (1.64%), *Veillonella* (1.43%), *Comamonas* (1.30%), *Pseudomonas* (1.24%), *Hyphomonas* (1.16%), *Novosphingobium* (1.13%), *Niabella* (1.11%), and *Alicycliphilus* (1.03%).

At the species level, a total of 7,023 species were annotated by metagenomic sequencing (Figure 1A). The predominant species were *Thauera phenylacetica* (8.62%), *Thauera unclassified* (5.81%), *Ottowia thiooxydans* (3.68%), *Nitrosomonas europaea* (2.62%), *Thauera aminoaromatica* (1.48%), *Citrobacter freundii* (1.16%), *Arenimonas malthae* (1.01%). These results indicated that the genus *Thauera* comprised the highest proportion of bacteria, with *O. thiooxydans* being the dominant species in the *Ottowia* genus, and *A. malthae* and *Arenimonas donghaiensis* being dominant species in the *Arenimonas* genus.

### 3.3 Composition and abundance of eukaryotes, archaea and viruses in the activated sludge

In the domain of eukaryotes, a total of eight phyla, 121 genera, and 159 species were detected in the activated sludge (Figure 1B). At the phylum level, Ascomycota (52.4%) was the most abundant phylum, followed by Basidiomycota (20.8%), Eukaryota noname (15.0%), Microsporidia (5.3%), Glomeromycota (3.1%), Blastocladiomycota (1.3%), Chytridiomycota (1.1%), and Cryptomycota (1.1%) in order of relative abundances. At the genus level, over nine genera, including *Mucor*, *Pseudogymnoascus*, *Drechslerella*, *Wallemia*, *Mortierella*, *Candida*, *Moniliophthora*, *Rhizophagus*, *Byssochlamys*, and *Rhizopus*, accounted for a large proportion in the activated sludge, with relative abundances ranging from 2.59 to 5.46%. Notably, *Pseudogymnoascus pannorum* (5.41%), *Drechslerella stenobrocha* (5.18%), *Mucor circinelloides* (4.15%), *Wallemia ichthyophaga* (3.91%), *Mortierella verticillata* (3.76%), *Moniliophthora roreri* (3.48%), *Rhizophagus irregularis* (3.06%), *Byssochlamys spectabilis* (2.62%), *Mitosporidium daphniae* (2.52%), *Candida maltosa* (2.40%), *Endocarpon pusillum* (2.33%), and *Puccinia graminis* (2.08%) were identified as dominant eukaryotic species, each with a relative abundance of ≥2%.

Similarly, the archaea domain included nine different phyla, in which Euryarchaeota (89.2%), Archaea noname (5.0%), Crenarchaeota (2.6%), and Thaumarchaeota (2.3%) were represented as the top four most abundant ones (Figure 1C). At the genus level, *Methanosaeta* was the largest genus, which accounted for 43.2%. The second largest genus was *Methanosarcina* (10.2%), followed by *Archaea noname* (5.0%). At the species level, a total of 232 species were annotated, in which the dominant species were *Methanosaeta concilii*, *Candidatus Methanoperedens nitroreducens*, and *Methanosarcina mazei*, which accounted for 41.8, 2.1, and 2.1%, respectively.

At the order level, viruses were dominated by Caudovirales (83.2%) in the activated sludge (Supplementary Figure S2D). At the genus level, the predominant viruses included *Podoviridae noname*, *N4likevirus*, and *Siphoviridae noname*, and the relative abundance of which were 22.3, 18.2, and 16.1%, respectively (Figure 1D). Additionally, a total of 559 species were annotated at the species level, in which the most abundant populations were *uncultured Mediterranean phage uvMED* (20.1%), *Ralstonia phage RSB3* (11.2%), *EBPR podovirus 3* (8.8%), and *Escherichia phage N4* (8.5%).

### 3.4 Distribution of genes associated with KEGG pathways in the activated sludge

**Figure 2:**
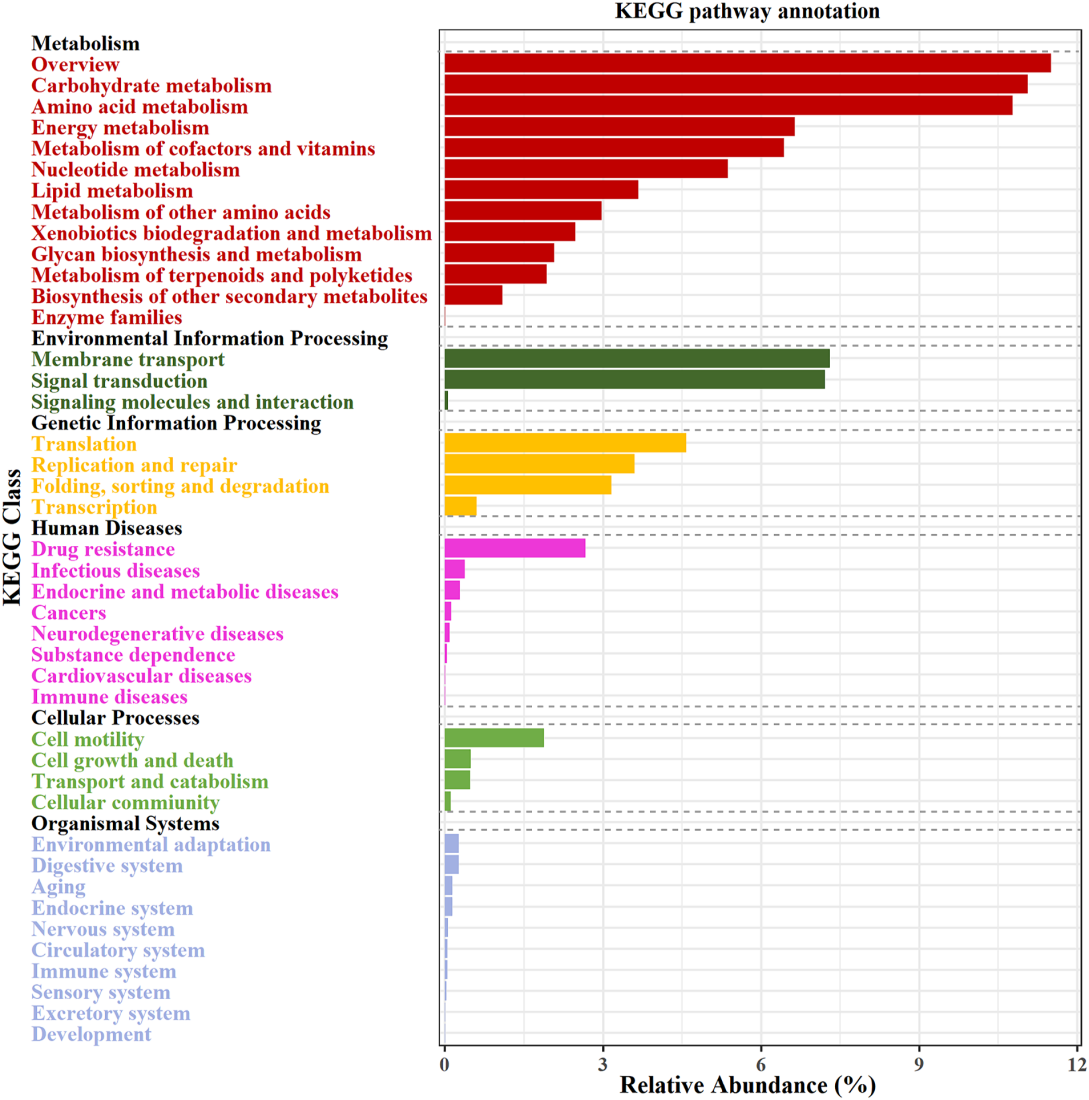
Potential function of genes detected in the activated sludge, annotated by KEGG database. The first level is displayed in black font, and the second level is displayed in color font.

To investigate the metabolic capabilities of activated sludge microbiome, genes were annotated and categorized according to the KEGG database. Figure 2 illustrates a visual distribution map of microbial functional metabolism in the activated sludge, derived from mapping metagenomic unigenes against the KEGG PATHWAY database using DIAMOND software. In total, 42 functional modules were identified as enriched, primarily in key KEGG pathways such as carbohydrate metabolism (11.06%), amino acid metabolism (10.78%), and membrane transport (7.30%).

**Figure 3:**
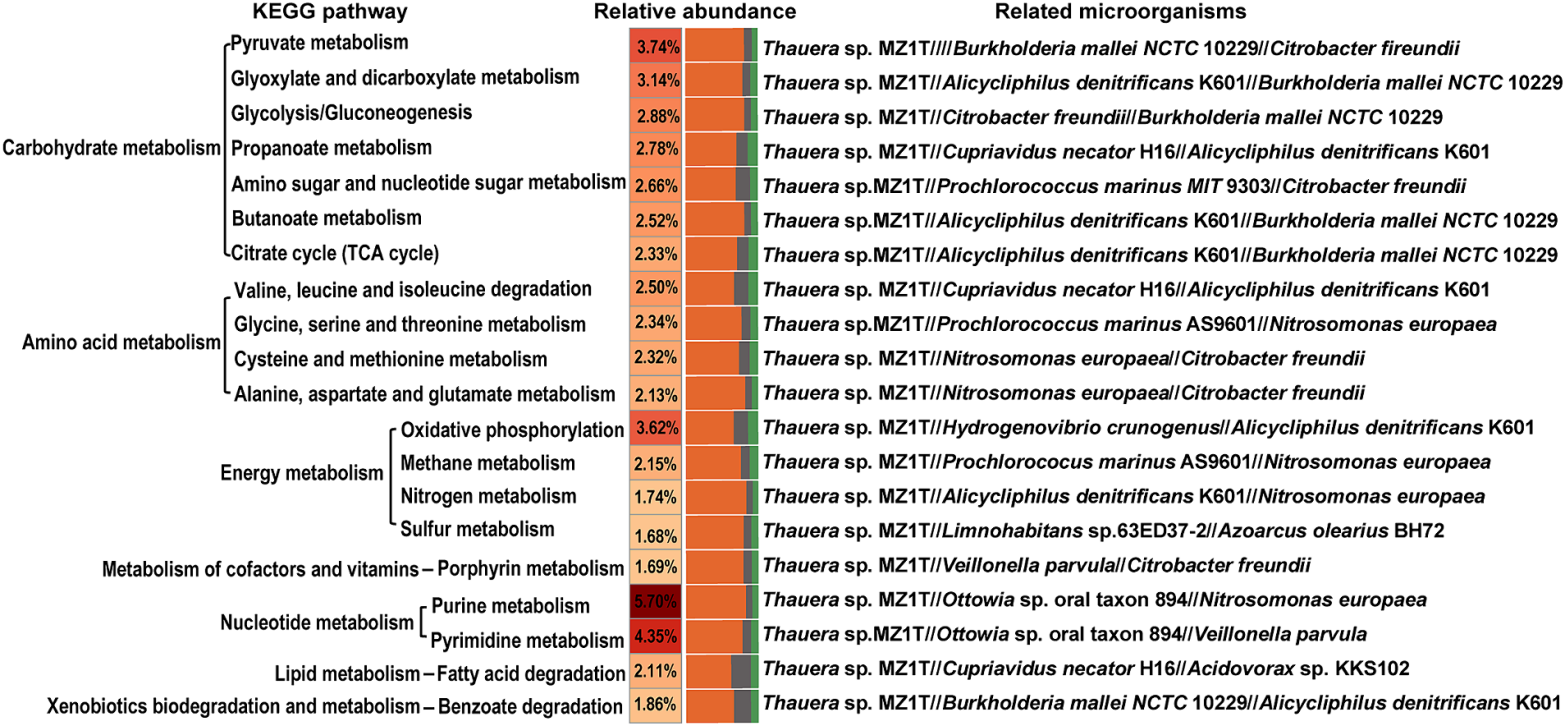
KEGG pathway analysis of the top 20 outcomes of related microorganisms. The color gradient is depicted on the left and reflects the relative abundance of metabolic pathways. The bar chart on the right represents the abundance of the top three bacterial species associated with each metabolic pathway.

The KEGG pathway enrichment analysis was performed as illustrated in Figure 3. The analysis highlights five metabolic pathways with notable relative abundances: purine metabolism (5.70%), pyrimidine metabolism (4.35%), pyruvate metabolism (3.74%), oxidative phosphorylation (3.62%), and glyoxylate and dicarboxylate metabolism (3.14%). Key microorganisms include: (1) Purine metabolism: *Thauera* sp. MZ1T, *Ottowia* sp. oral taxon 894, *N. europaea*, among others; (2) Pyrimidine metabolism: *Thauera* sp. MZ1T, *Ottowia* sp. oral taxon 894, *Veillonella parvula*, and other potential strains; (3) Pyruvate metabolism: *Thauera* sp. MZ1T, *Burkholderia mallei* NCTC 10229, *C. freundii*, and additional strains; (4) Oxidative phosphorylation: *Thauera* sp. MZ1T, *Hydrogenovibrio crunogenus*, *Alicycliphilus denitrificans* K601, among others; (5) Glyoxylate and dicarboxylate metabolism: *Thauera* sp. MZ1T, *A. denitrificans* K601, *B. mallei* NCTC 10229, and other strains.

The analysis further revealed microorganism’s participation in the nitrogen metabolism pathway, involving *Thauera* sp. MZ1T, *A. denitrificans* K601, *N. europaea*, and other potential strains, contributing to approximately 1.74% of the overall metabolic processes. Notably, the fatty acid degradation pathway accounted for 2.11% of the metabolic pathways annotated through the KEGG database, and it was mainly related to *Thauera* sp. MZ1T, *Cupriavidus necator* H16, and *Acidovorax* sp. KKS102.

### 3.5 Functional genes and microorganisms associated with nitrogen metabolism

**Figure 4:**
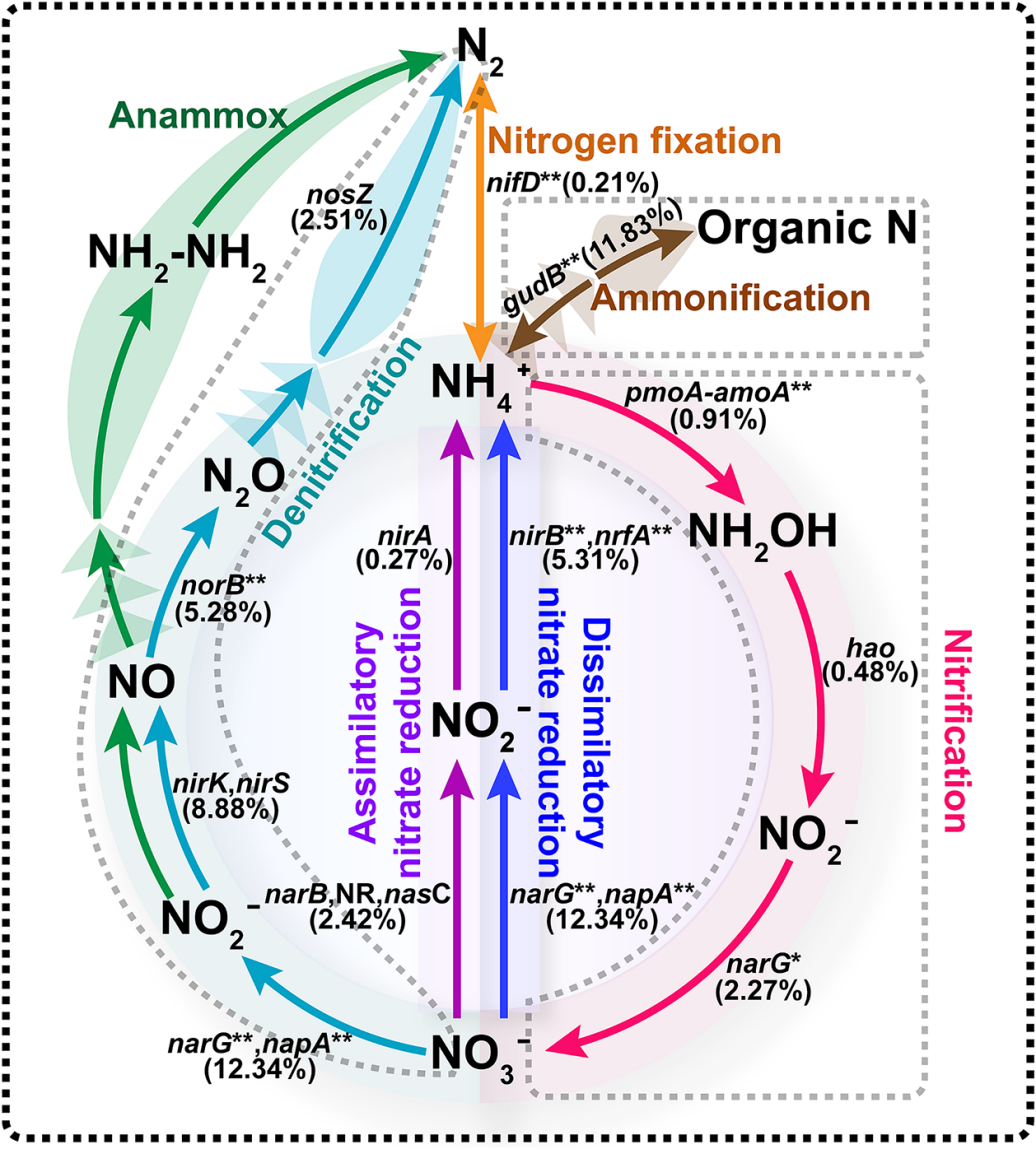
Nitrogen metabolism pathway of activated sludge. Functional genes involved in different processes are represented in different colors, with the abundance of the corresponding gene in parentheses. The symbol “**” represented the omitted genes. Ammonification: *gudB*/*GLUD1_2*/*E1.4.1.4*/*GDH2*, glutamate dehydrogenase. Nitrification: *pmoA-amoA*/*pmoB-amoB*/*pmoC-amoC*, methane/ammonia monooxygenase; *hao*, hydroxylamine dehydrogenase; *narG*/*narH* nitrate reductase/nitrite oxidoreductase. Denitrification: *narG/narH/narl/napA/napB*, nitrate reductase; *nirK/nirS*, nitrite reductase; *norB/norC*, nitric oxide reductase; *nosZ*, nitrous-oxide reductase. Assimilatory nitrate reduction: *narB*, ferredoxin-nitrate reductase; *nasC*, assimilatory nitrate reductase; *nirA*, ferredoxin-nitrite reductase. Dssimilatory nitrate reduction: *narG/narH/narl/napA/napB*, nitrate reductase; *nirB/nirD/nrfA/nrfH*, nitrite reductase. Nitrogen fixation: *nifD/nifK/nifH*, nitrogenase.

Biological nitrogen removal was one of the most important functions of activated sludge. In this study, we identified 6,630 genes involved in the nitrogen metabolism pathway. Figure 4 demonstrated that the microorganisms in the activated sludge participate in several nitrogen-related processes, including nitrogen fixation, ammonification, nitrification, denitrification, assimilative and dissimilative nitrate reduction. Notably, there was a high abundance of genes linked to the denitrification process. During denitrification, nitrate is successively converted into nitrite and nitric oxide by nitrate reductase (*narG, napA*, 12.34%) and nitrite reductase (*nirK*, *nirS*, 8.88%), respectively. Subsequently, nitric oxide is converted into nitrous oxide and then nitrogen by nitric oxide reductase (*norB*, 5.28%) and nitrous oxide reductase (*nosZ*, 2.51%). The gene relative abundance of nitrate reductase (*narG*, *napA*, 12.34%) was significantly higher compared to other denitrification enzymes, whereas nitrous oxide reductase (*nosZ*, 2.51%) showed lower abundance. Additionally, the functional gene *gudB*, encoding glutamate dehydrogenase, was the second most abundant, involved in the conversion of organic nitrogen to inorganic nitrogen. However, our metagenome analysis in the activated sludge did not identify any functional genes or essential enzymes associated with the anammox pathway.

Additionally, the principal microorganisms involved in biological nitrogen removal were examined (Table 2). A total of 616 species were linked to nitrogen metabolism pathways. The 10 species with the highest abundance were *Thauera* sp. MZ1T (25.15%), *A. denitrificans* K601 (2.49%), *N. europaea* (2.18%), *Ottowia* sp. oral taxon 894 (1.67%), *C. freundii* (1.66%), *Azoarcus olearius* BH72 (119.64), *V. parvula* (1.36%), *Stenotrophomonas acidaminiphila* (1.21%), *Pseudoxanthomonas suwonensis* 11-1 (1.03%), and *Acidovorax* sp. JS42 (0.95%). Among these, six species were specifically noted for their involvement in the denitrification process: *Thauera* sp. MZ1T, *A. denitrificans* K601, *Ottowia* sp. oral taxon 894, *S. acidaminiphila*, *P. suwonensis* 11-1, and *Acidovorax* sp. JS42. Notably, *S. acidaminiphila* and *P. suwonensis* 11-1, despite not being linked to the *nosZ* gene during denitrification, are known to produce the greenhouse gas N_2_O. Furthermore, *N. europaea* plays a crucial role in converting ammonia nitrogen to nitric nitrogen, while *A. olearius* BH72 is involved in nitrogen fixation.

**Table 2:** The key microorganisms detected in the metagenomic sequences of activated sludge involved in nitrogen cycle.

Species	Relative abundance	Functions
*Thauera* sp. MZ1T	25.15%	Denitrification, assimilatory nitrate reduction
*Alicycliphilus denitrificans* K601	2.49%	Denitrification, assimilatory nitrate reduction
*Nitrosomonas europaea*	2.18%	Nitrification
*Ottowia* sp. oral taxon 894	1.67%	Denitrification
*Citrobacter freundii*	1.66%	Dissimilatory nitrate reduction
*Azoarcus olearius* BH72	1.59%	Nitrogen fixation, dissimilatory nitrate reduction
*Veillonella parvula*	1.36%	Nitrogen metabolism
*Stenotrophomonas acidaminiphila*	1.21%	Denitrification (N_2_O)
*Pseudoxanthomonas suwonensis* 11-1	1.03%	Denitrification (N_2_O), assimilatory nitrate reduction
*Acidovorax* sp. JS42	0.95%	Denitrification, assimilatory nitrate reduction

The second column represents the abundance of nitrogen metabolic pathways in species, and the third column represents the specific function of strains in the process of nitrogen metabolism.

### 3.6 Analysis of functional genes, key enzymes, and microorganisms related to lipid metabolism

**Figure 5:**
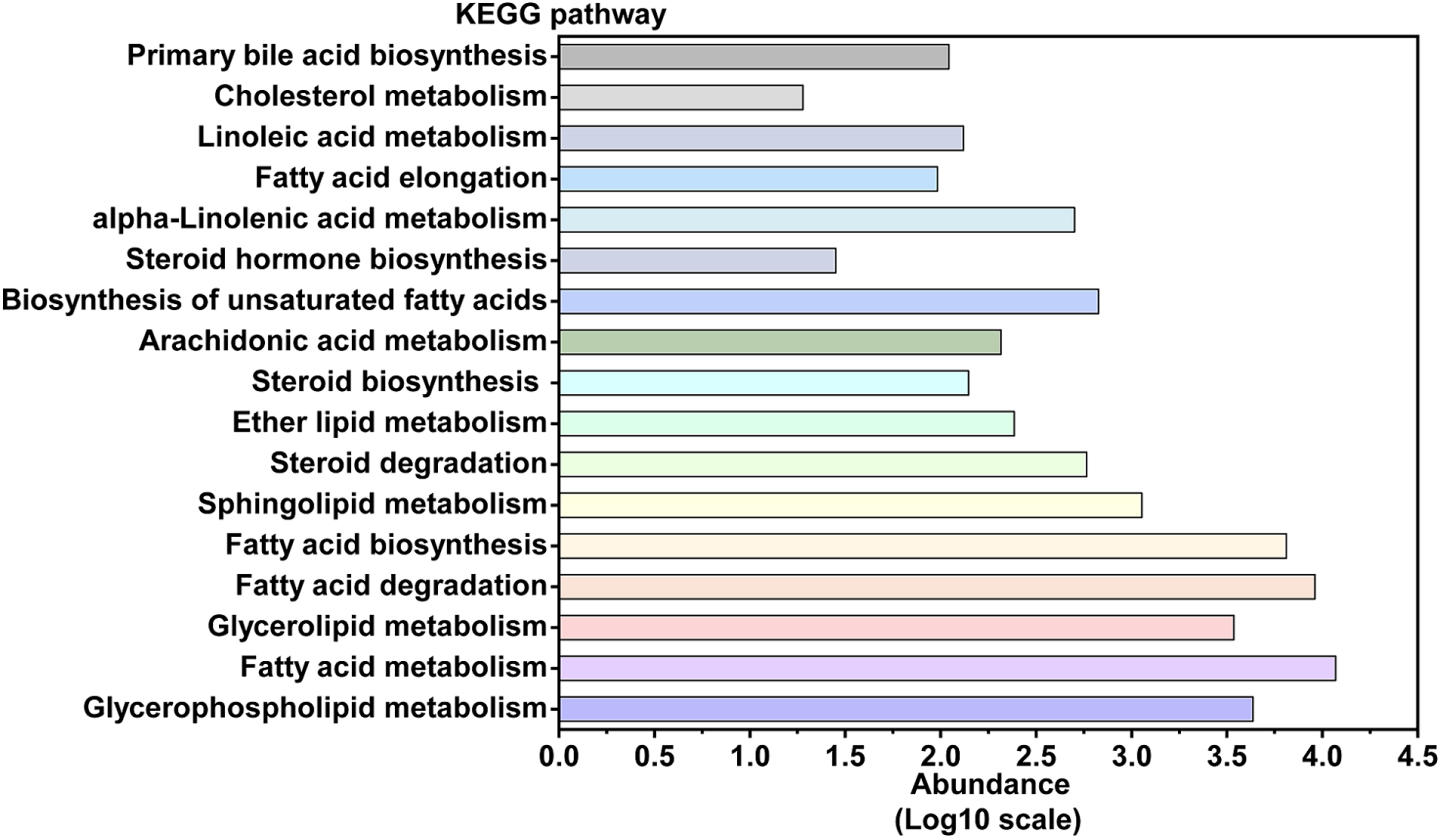
The abundance of each pathway in the lipid metabolism, annotated by KEGG ORTHOLOGY database.

**Figure 6:**
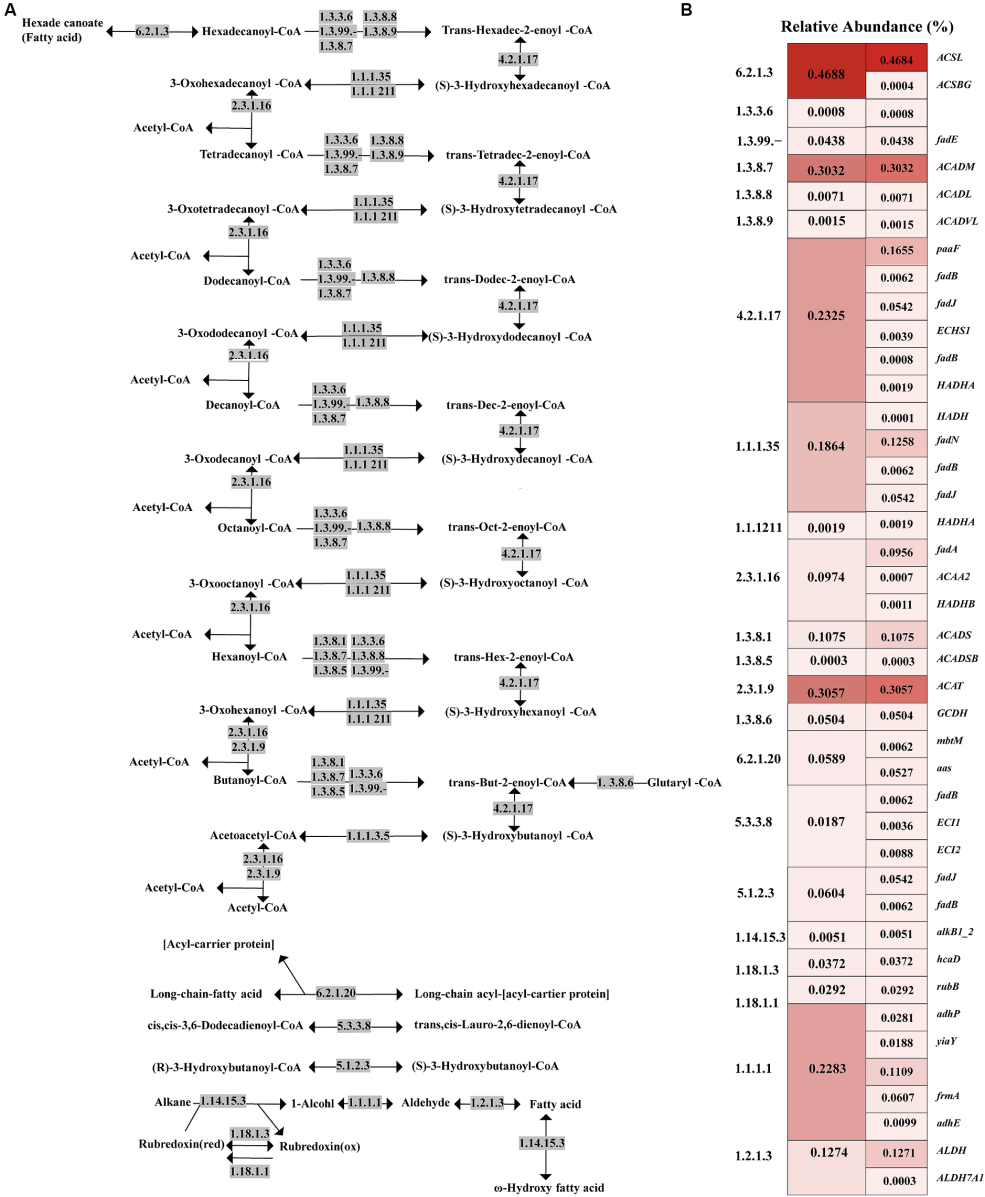
Schematic diagram of fatty acid degradation pathways and the relative abundance of enzymes involved in each step. **(A)** The degradation process of saturated fatty acids under the action of key enzymes is mainly described, and other fatty acid degradation methods are briefly mentioned in the bottom half of the figure; **(B)** Heat map of relative abundance of functional genes and their encoded enzymes in fatty acid degradation.

Given that tuna processing wastewater contains a significant amount of lipids and lipid-like molecules, lipid metabolism is a crucial pathway for focus within the activated sludge microbial community (Chipasa and Medrzycka, 2008). Figure 5 illustrates that, according to the KO database comparison, the most abundant metabolic pathways in the activated sludge are fatty acid metabolism, fatty acid degradation, and fatty acid biosynthesis. To further explore the fatty acid degradation process in the activated sludge, a schematic diagram of this pathway, including the involved functional genes and key enzymes, is presented in Figure 6 and Supplementary Table S3. The gene *ACSL*, which encodes long-chain acyl-CoA synthase (EC: 6.2.1.3), exhibited the highest relative abundance at 0.4684%. The gene *ACAT*, responsible for acetyl-CoA C-acetyltransferase (EC: 2.3.1.9), was the second most abundant, accounting for 0.3057%. Additionally, acyl-CoA dehydrogenase (EC: 1.3.8.7), encoded by the gene *ACADM*, was also significant in the oxidation of long-chain fatty acids with a relative abundance of 0.3020%. Enoyl-CoA hydratase (EC: 4.2.1.17) showed a higher proportion with a relative abundance of 0.2325%, with the gene *paaF* accounting for the largest proportion among the genes encoding this enzyme.

Additionally, the results indicated a total of 733 relevant species were involved in fatty acid degradation. Top 10 species with the highest abundance were *T. aminoaromatica* (14.27%), *C. necator* H16 (6.75%), *Desulfotalea psychrophile* (1.52%), *A. denitrificans* K601 (1.39%), *Hyphomonas neptunium* (1.27%), *Nitrosomonas eutropha* (1.08%), *C. freundii* (1.04%), *Pseudomonas putida* DOT-T1E (1.01%), *Delftia acidovorans* (0.96%), and *N. europaea* (0.95%) (Table 3). The functional analysis revealed that *T. aminoaromatica*, *C. necator* H16, *C. freundii*, *P. putida* DOT-T1E and *D. acidovorans* possessed comprehensive degradation capabilities for unsaturated fatty acids, encompassing both acyl-CoA synthesis and beta-oxidation pathways, while *D. psychrophile*, *A. denitrificans* K601, *N. eutropha* and *N. europaea* were limited to participating in selective degradation processes of fatty acids. Besides, *H. neptunium* exhibited exclusive competence in the full degradation of hexanoyl-CoA and butanoyl-CoA.

**Table 3:** The key microorganisms detected in the metagenomic sequences of activated sludge involved in fatty acid degradation.

Species	Relative abundance	Functions
*Thauera aminoaromatica*	14.27%	acyl-CoA synthesis, beta-Oxidation
*Cupriavidus necator* H16	5.75%	acyl-CoA synthesis, beta-Oxidation
*Desulfotalea psychrophila*	1.52%	acyl-CoA synthesis
*Alicycliphilus denitrificans* K601	1.39%	acyl-CoA synthesis
*Hyphomonas neptunium*	1.27%	beta-Oxidation (Hexanoyl-CoA, Butanoyl-CoA)
*Nitrosomonas eutropha*	1.08%	acyl-CoA synthesis
*Citrobacter freundii*	1.04%	acyl-CoA synthesis, beta-Oxidation
*Pseudomonas putida* DOT-T1E	1.01%	acyl-CoA synthesis, beta-Oxidation
*Delftia acidovorans*	0.96%	acyl-CoA synthesis, beta-Oxidation
*Nitrosomonas europaea*	0.95%	acyl-CoA synthesis

The second column represents the abundance of fatty acid degradation pathway in species, and the third column represents the specific function of strains in the process of fatty acid degradation.

## 4 Discussion

### 4.1 Composition of metagenome communities in the activated sludge

It is well-known that activated sludge microbial communities play a crucial role in the degradation and removal of pollutants in wastewater treatment plants (Perez-Esteban et al., 2022). The performance of various activated sludge systems is determined by the structure, variety, and function of these microbial communities (Wang et al., 2024). In contrast to typical municipal wastewater, tuna processing wastewater contains higher levels of fat and protein, classifying it as organic wastewater with elevated concentrations of lipids and nitrogen. To elucidate the role of microbial communities in degrading organic pollutants in the activated sludge, we analyzed the microbial community structure and function in the activated sludge from food processing wastewater, characterized by high concentrations of lipids and nitrogen (Supplementary Figure S1). These findings may illuminate the unique mechanisms through which activated sludge from food processing facilities contributes to wastewater treatment.

The results revealed that the activated sludge had a remarkably extensive range of variety, as evidenced by the identification of 1,880 genus and 7,974 species in total. Consistent with the previous reports, it was shown that the bacteria were the most predominant and the prevailing phylum was proteobacteria (Figure 1A), representing the highest proportion of activated sludge (Thomsen et al., 2007; More et al., 2014). At the genus level, the prevailing bacteria that pertain to the effectiveness of sewage treatment were primarily associated with the phylum Proteobacteria, such as *Thauera*, *Arenimonas*, *Nitrosomonas*, *Niabella*, which were typical genus denitrifying bacteria (*DNB*), anaerobic ammonia-oxidizing bacteria (*anAOB*), ammonia-oxidizing bacteria (*AOB*) and nitrite-oxidizing bacteria (*NOB*) (Fumasoli et al., 2015; Ren et al., 2021; Zhang et al., 2023). Among them, *Thauera* (24.6%) was the dominant genus in Proteobacteria, belonging to a Gram-negative bacterium in β-proteobacteria. As one of the core members in the activated sludge, *Thauera* played an important role in the process of nitrogen and phosphorus removal in wastewater, showing a strong denitrification ability in aerobic/anoxic experiments (Ren et al., 2021). In the present study, both *Ottowia* and *Arenimonas* were unique dominant genera in the activated sludge (Figure 1A), which were not detected among the top 10 dominant bacterial genera in a common effluent treatment plant in South India and a municipal wastewater treatment plant (More et al., 2014; Guo et al., 2017). *Ottowia* has been frequently found a predominant bacterial genus in petroleum refinery wastewater treatment plants, along with *Thauera*, which have been isolated and identified in the activated sludge contaminated with phenol, benzene, toluene, ethylbenzene, and xylene (Kim et al., 2020). *Arenimonas*, in addition to being typical anaerobic ammonia oxidizing bacteria, is often found in oil-contaminated soil and may have the potential for oil degradation (Young et al., 2007; Gray et al., 2022).

At the species level, the dominant strain *T. phenylacetica* was considered to be a denitrifier (Figure 1A), which took the nitrate, nitrite and oxygen as electron acceptors (Mechichi et al., 2002). However, unlike *T. aminoaromatica*, which can completely convert nitrate to nitrogen, *T. phenylacetica* lacks the *nosZ* gene that plays a key role in converting all nitrate to N_2_O (Bakken et al., 2012). Additionally, it was reported that the denitrifying bacterium was specialized in degrading phenylacetic acid, and it had been lately found to be a metabolite of phenylalanine, which was associated with lipid metabolism (Rajta et al., 2020). In this study, *Ignavibacterium album* and *N. europaea* of the microorganisms detected in the activated sludge were also the dominant strains in the sequencing batch biofilm reactor with the removal rates of NH_4_^+^–N and TN of 99.4 and 90.5%, respectively (Zhang et al., 2014). Notably, *O. thiooxydans* was also a dominant strain in this study. Previous studies have shown that *O. thiooxydans* is also known as a sulfur-oxidizing bacteria with the ability to oxidize sulfide except for the removal of nitrogen (Spring et al., 2004). In addition, another dominant strain *A. malthae* in this study may have the ability to utilize organic acids as sufficient organic carbon substrate (Young et al., 2007). Taken together, the taxonomy of bacteria above indicated that a variety of functional microorganisms originating from the activated sludge could play crucial roles in nitrogen and lipid metabolism.

Previous research on the microbial composition of activated sludge predominantly focuses on bacteria, neglecting the significant roles played by fungal archaea and even viruses in this complex ecosystem. It is reported that fungi can secrete enzymes to exert their functions in the external environment (Alves et al., 2002). In the activated sludge microbiota, the distribution of fungi is relatively balanced (Figure 1B). Research had shown that the dominant genus *Mucor* had the ability to produce protease and lipase, and showed strong protein and fat degradation ability (Alves et al., 2002). *Mucor circinelloides* detected in the activated sludge was an oleaginous filamentous fungus, serving as a model organism for studying lipid metabolism in oil-rich microorganisms (Vellanki et al., 2018; Li et al., 2022). Another fungus identified, *Wallemia ichthyophaga*, represents the most halophilic eukaryotes described to date, which can adapt to high-glucose environments (Kralj Kunčič et al., 2013).

Due to the nascent stage of detection and characterization studies on archaea, the role of archaeal communities in the activated sludge is often overlooked. *Methanosaeta*, as the predominant genus in the activated sludge, can contribute significantly to global methane production on the planet (Ju et al., 2014). The most abundant archaeal species, *M. concilii*, exhibited high tolerance to concentrations of long-chain fatty acids (Figure 1C) (Silva et al., 2016). Furthermore, it was found that *Ca.* M. nitroreducens was an archaeon that could combine anaerobic methane oxidation with nitrate reduction (Guerrero-Cruz et al., 2018). It has also been reported that *Ca.* M. nitroreducens was detected in wastewater treatment plants in both northern and southern regions of China (Xu et al., 2020).

It has been previously established that viruses present in the activated sludge have auxiliary metabolic functions that may influence the element cycling and chemical composition of wastewater (Yuan and Ju, 2023). In this study, the majority of identified viral phages belonged to the order Caudovirales, which was double-stranded DNA virus, including *Podoviridae*, *Siphoviridae*, and *Myoviridae* (Supplementary Figure S2D). These phages exhibit a wide host range and play a crucial role in regulating bacterial populations in diverse ecosystems; however, the specific mechanisms of their impact are still unclear and require further exploration.

### 4.2 Nitrogen removal potential of microbial communities in activated sludge

After determining functional genes in the activated sludge, the KEGG enrichment pathway analysis was performed, showing that these unigenes were mainly enriched in the pathways of carbohydrate degradation ability and amino acid metabolism (Figure 2). In conjunction with such microbial pathways of regular growth metabolism, the presence of sequences associated with nitrogen and lipid metabolisms is crucial for the efficacy of wastewater treatment plant. It is well known that microbial nitrogen removal is one of the most important contributions in the activated sludge process (Ghafari et al., 2008; Joicy et al., 2019). Scholars had explored the functional genes and microorganisms related to nitrogen metabolism to evaluate the ability of activated sludge, and discovered some key microorganisms, e.g., *Nitrosomonas* and *Nitrosospira* (Tian et al., 2015). In this study, the functional genes and the key microorganisms in the process of biological denitrification were analyzed to explore the unique structure of the denitrification microflora of activated sludge in food processing wastewater. Metagenomic data analysis indicated that the main mechanism responsible for nitrogen removal in the activated sludge microbial communities was involved in the combined process of nitrification and denitrification (Figure 4). Functionally relevant genes associated with denitrification were highly enriched, and key microbial species involved in this process included *Thauera* sp. MZ1T, *A. denitrificans* K601, *Ottowia* sp. oral taxon 894, *S. acidaminiphila*, *P. suwonensis* 11-1, *Acidovorax* sp. JS42, among others (Table 2). These microorganisms can effectively reduce nitrate to nitrogen gas. However, it is worth noting that not all microorganisms possess a complete set of denitrification functional genes. For instance, *S. acidaminiphila* and *P. suwonensis* 11-1 lack the *nosZ* gene, resulting in the generation of only the greenhouse gas N_2_O during the denitrification process (Guo et al., 2017; Cao et al., 2024). In contrast, the abundance of functional genes in the denitrification process of activated sludge in large municipal wastewater treatment plants is roughly the same and maintained at a high abundance level (Guo et al., 2017). In addition, *N. europaea* is known as the main ammonia-oxidizing bacterium, which is responsible for converting ammonia nitrogen into nitric nitrogen, and *A. oleari*us BH72 is involved in the process of nitrogen fixation (Table 2; Padrao et al., 2019; Mani and Reinhold-Hurek, 2021). Studies have shown that *A. oleariu*s BH72 is an endophyte capable of biofixing nitrogen (BNF) and supplying nitrogen to host plants (Shanmugam and Barbara, 2021). This strain has been shown to colonize rice roots. However, the metagenomic (Shanmugam, 2021) analysis of the sludge did not reveal the presence of any key enzymes associated with the anammox pathway (Figure 4).

### 4.3 Biodegradation of related fatty acids by activated sludge microorganism

In addition to being closely related to nitrogen metabolism, the wastewater generated from tuna processing is rich in lipids, and thus it might be necessary to perform a thorough investigation into the lipid metabolism pathway within activated sludge microorganisms. To assess the efficacy of activated sludge in decomposing lipids present, our study undertook a comprehensive exploration of microbial lipid metabolism processes (Figure 5). The present research observed a relative abundance of the fatty acid degradation pathway at 2.11%, which was found to be higher than that observed in a typical municipal sewage treatment plant (Guo et al., 2017; Figure 3). According to previous reports, lipid molecules were first hydrolyzed into fatty acids by extracellular lipase enzymes from microorganisms. Subsequently, these fatty acids were transported into microorganism cells for further dehydrogenation and hydration process facilitated by β-oxidation enzymes (Chipasa and Medrzycka, 2008). Hence, the degradation process proceeded through β-oxidation starting from the end of the fatty acid chain, resulting in a gradual decrease in the carbon number of the substance (Beldean-Galea et al., 2013). Functional analysis revealed the presence of key enzymes involved in lipid degradation process in the activated sludge, with the long-chain acyl-CoA synthase encoded by the gene *ACSL* exhibiting the highest abundance (Figure 6). Previous research revealed that this enzyme played a crucial role in activating fatty acids by forming fatty acyl-CoA which was an essential step in fatty acid oxidation (Quan et al., 2021). Additionally, acyl-CoA dehydrogenase and enoyl-CoA hydratase were found to be highly enriched, and they functioned as the dehydrogenation and hydration, respectively, during the β-oxidation of fatty acids (Figure 6).

Metagenomic data results indicated a diversity of microorganisms associated with lipid degradation (Table 3). It was reported that the genome of the related strain *C. necator* H16 contained many homologs of genes encoding β-oxidases related to the beta-Oxidation reactions that were involved in fatty acid degradation, suggesting that *C. necator* H16 was considered to be a strong candidate for the production of specific chain-length organic acids (Strittmatter et al., 2022). In addition to its denitrification capability, *A. denitrificans* K601 exhibited the ability to utilize cyclic lipids as a sole carbon source and organic acids, e.g., lactic acid, acetic acid, and propionic acid, as alternative carbon sources (Oosterkamp et al., 2011). The investigation of this strain can hold a significant implication for comprehending the degradation process of cyclic lipids in the environment and nitrogen cycling within ecosystems (Oosterkamp et al., 2011). *P. putida* DOT-T1E, a Gram-negative rod-shaped bacterium, exhibits the ability to metabolize aromatic compounds and demonstrates high tolerance and degradation capability toward organic pollutants in the environment (García et al., 2010). The metagenomic results of this study have shown that *P. putida* DOT-T1E possess intact beta-oxidation functional genes (Table 3). Therefore, it is crucial to further investigate its fatty acid degradation mechanism and explore strategies for enhancing its degradation ability through strain modification. Moreover, prior research showed that *D. acidovorans* identified in the soil around Shiraz refinery could degrade aniline and had the ability to promote plant growth and degrade organic pollutants (Urata et al., 2004). Above all, this study can facilitate the exploration of exceptionally efficient strains associated with lipid degradation.

Comprehensive analysis revealed that the metagenomics of activated sludge possessed a distinctive metabolic signature, which was closely related to the presence of some special microbial communities. It is noteworthy that the dominant microorganisms are crucial for the removal of complex organic compounds rich in lipids and nitrogen in the activated sludge. Thereby, the omics information can serve as a reference for optimizing process conditions and operational parameters for the treatment of wastewater from food processing. Concomitantly, the search for efficient degrading bacteria can be applied for the biological strengthening of activated sludge to enhance the performance of activated sludge in treating special wastewater. For instance, a lipid degrading microbe consortium was assembled to improve the performance of activated sludge on cooking wastewater purification (Wang et al., 2022). However, although the roles and functions of dominant microorganisms get considerable attention, the microbial systems exhibit high diversity and there is a lack of research on low-abundance microorganisms. For this reason, it is very necessary for further analyzing the overall microbial community structure by increasing the sample numbers and using comprehensive omics techniques. In the future, the more profound research on these aspects may provide a new perspective for underlying the important role of microorganisms in the activated sludge.

## 5 Conclusion

In this study, metagenomic sequencing was utilized to investigate the core microorganisms in the activated sludge for treating tuna processing wastewater. The results revealed that the microbial community in the activated sludge from tuna processing wastewater exhibited high microbial diversity and a nitrogen removal pathway. Additionally, the microbial community included various dominant strains associated with fatty acid degradation and sulfur metabolism. Correlative functional analysis elucidated the metabolic processes of the sludge and its associated microorganisms, with a particular focus on bacteria involved in nitrogen and lipid metabolism. This study revealed the microbial composition and related metabolic pathways in the activated sludge, which could contribute to expanding our understanding the role of core microorganisms in the activated sludge for treating tuna processing wastewater.

## Data availability statement

The datasets presented in this study can be found in online repositories. The names of the repository/repositories and accession number(s) can be found in the article/Supplementary material.

## Author contributions

ZZ: Conceptualization, Data curation, Formal analysis, Methodology, Writing – original draft, Writing – review & editing. CL: Data curation, Formal analysis, Writing – original draft. YC: Data curation, Formal analysis, Writing – original draft. TM: Conceptualization, Formal analysis, Investigation, Methodology, Writing – original draft, Writing – review & editing. LJ: Formal analysis, Writing – review & editing. FK: Formal analysis, Writing – review & editing. XS: Conceptualization, Data curation, Methodology, Writing – original draft. JX: Conceptualization, Data curation, Formal analysis, Funding acquisition, Investigation, Methodology, Writing – original draft, Writing – review & editing.
