# Supplementary material for: Revealing the functional potential of microbial community of activated sludge for treating tuna processing wastewater through metagenomic analysis
Source: Front Microbiol. 2024 Jul 19;15:1430199. doi: 10.3389/fmicb.2024.1430199 (PMC11294940; doi:10.3389/fmicb.2024.1430199)
Supplement: Supplementary file 1 [file Data_Sheet_1.docx]

Supplementary Material

Revealing the Composition and Diversity of Activated Sludge in Seafood Processing Wastewater through Metagenomic Analysis

Zhangyi Zheng^1,2^†, Changyu Liao^1^†, Yubin Chen^1,2^, Tinghong Ming^1,2*^, Lefei Jiao^1,2^, Fei Kong^1,2^, Xiurong Su^1,2^, Jiajie Xu^1,2*^

^1^School of Marine Science, Ningbo University, Ningbo, Zhejiang, China

^2^Microbial Development and Metabolic Engineering Laboratory, Ningbo University, Ningbo, Zhejiang, China

†These authors contributed equally to this work.

***Correspondence:**Tinghong Ming; Jiajie Xu

mingtinghong@nbu.edu.cn; xujiajie@nbu.edu.cn

# Supplementary Tables

**Table S1** **The components of activated sludge.**

**Table S2** **Water quality test result.** The sample was taken from the secondary settling tank.

**Table S3 Relative abundance of key functional genes and enzymes in fatty acid degradation process.** The enzymes encoded by the second column of genes are shown in the first and last columns, and the third column indicates the relative abundance of functional genes.

# Supplementary Figures

**Figure S1** The krona circles from the inside out represent different classification levels (Kingdom, Phylum, Class, Order, Family, Genus and Species); The size of the fan represents the relative proportions of different taxonomic levels.

**Figure S2** Microbial community composition of four kingdoms at the class, order and family levels. **(A)** Bacteria kingdom; **(B)** Eukaryota kingdom; **(C)** Archaea kingdom; **(D)** Viruses kingdom.


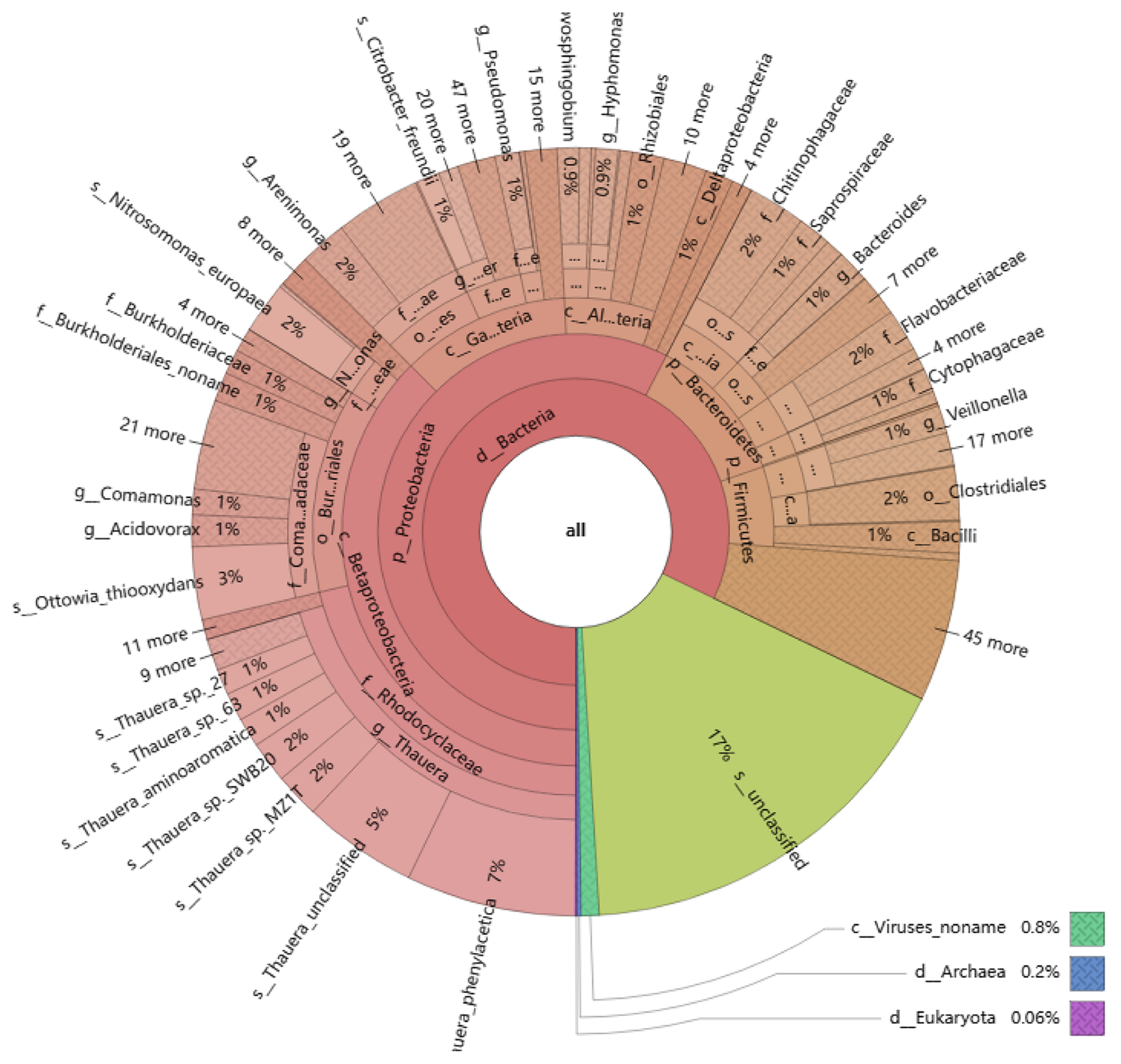


**Figure S1**


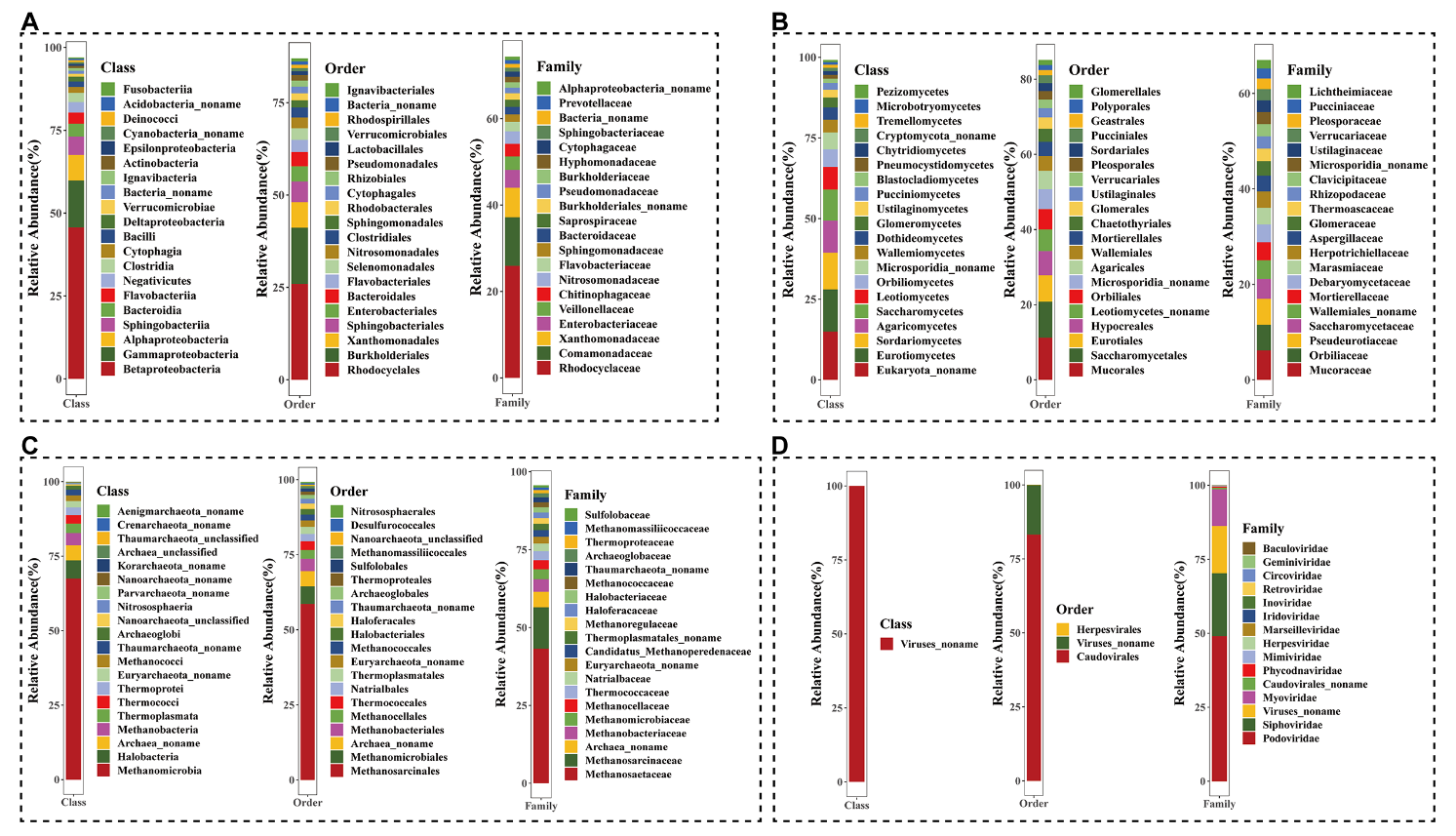


**Figure S2**
