# Supplementary figures and images for: Revealing the functional potential of microbial community of activated sludge for treating tuna processing wastewater through metagenomic analysis
Source: Front Microbiol. 2024 Jul 19;15:1430199. doi: 10.3389/fmicb.2024.1430199 (PMC11294940; doi:10.3389/fmicb.2024.1430199)

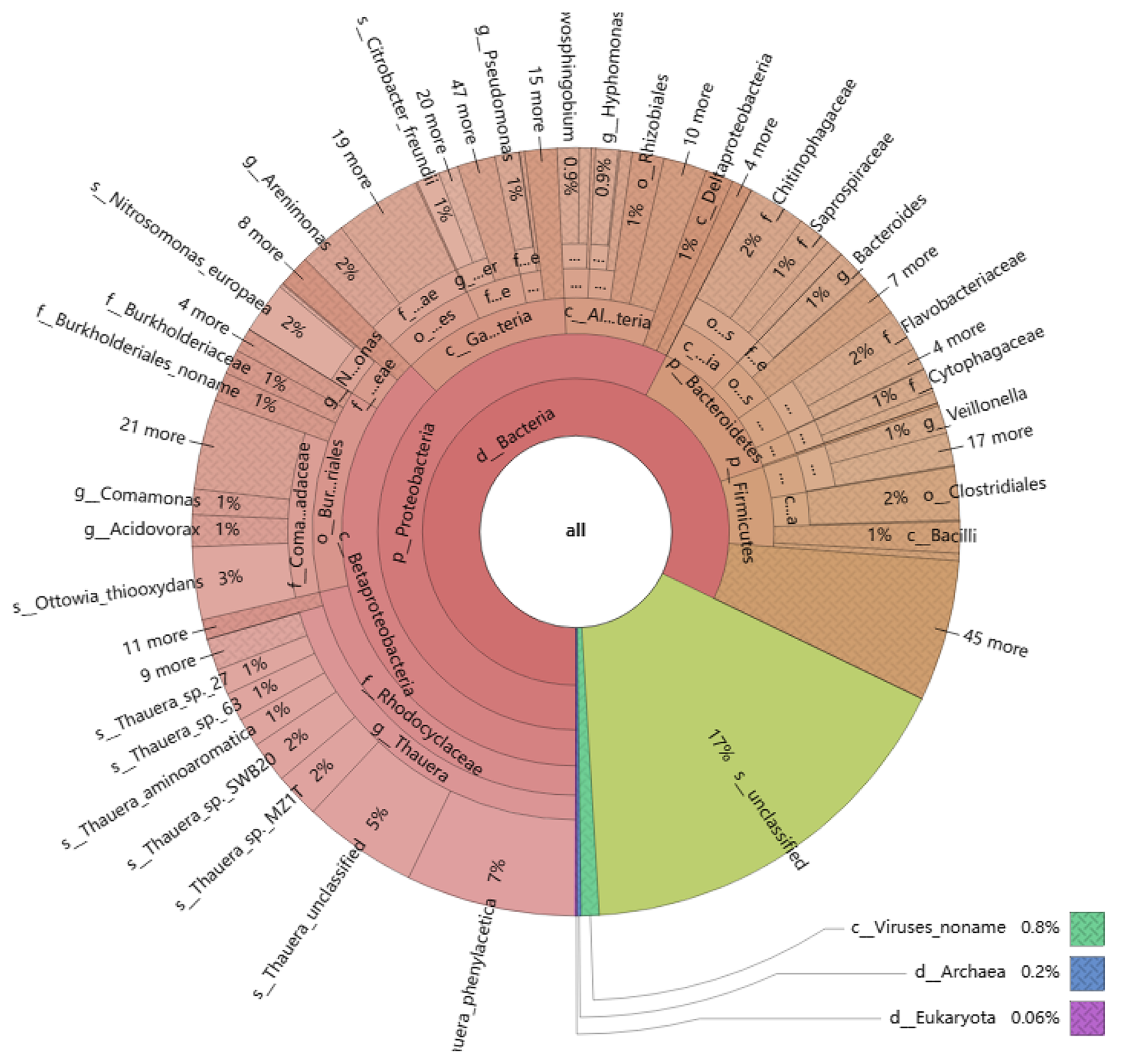

Supplement: Supplementary file 2 [file Image_1.TIF]

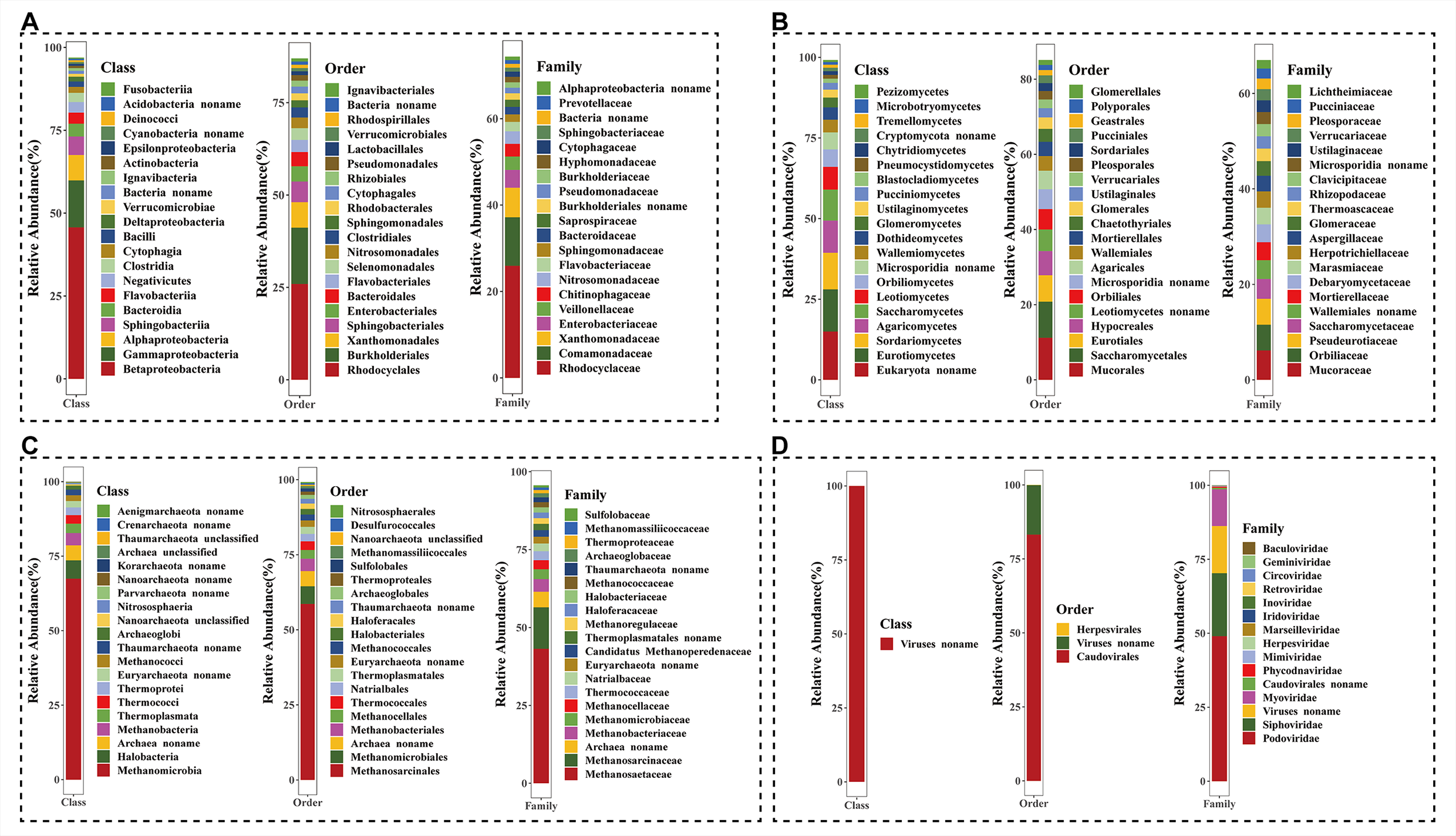

Supplement: Supplementary file 3 [file Image_2.TIF]
