# Supplementary material for: Revealing the functional potential of microbial community of activated sludge for treating tuna processing wastewater through metagenomic analysis
Source: Front Microbiol. 2024 Jul 19;15:1430199. doi: 10.3389/fmicb.2024.1430199 (PMC11294940; doi:10.3389/fmicb.2024.1430199)
Supplement: Supplementary file 4 [file Table_1.DOCX]

**Table S1** **The components of activated sludge.**

| Activated sludge index | Testing value |
| --- | --- |
| Lead (mg/kg) | 3.5 |
| Cadmium (mg/kg) | 5.9 |
| Aluminum (mg/kg) | 6.05×10^4^ |
| Arsenic (mg/kg) | 25 |
| Organic matter (%) | 34.8 |
