# Supplementary material for: Revealing the functional potential of microbial community of activated sludge for treating tuna processing wastewater through metagenomic analysis
Source: Front Microbiol. 2024 Jul 19;15:1430199. doi: 10.3389/fmicb.2024.1430199 (PMC11294940; doi:10.3389/fmicb.2024.1430199)
Supplement: Supplementary file 5 [file Table_2.DOCX]

**Table S2** **Water quality test result.** The sample was taken from the secondary settling tank.

| **Water quality index** | **Secondary sedimentation tank** |
| --- | --- |
| Ammonium-N (mg/L) | 35.67 ± 3.06 |
| Nitrite-N (mg/L) | < 0.015 |
| Nitrate-N (mg/L) | 0.45 ± 0.13 |
| Salinity (ppt) | 2.21 ± 0.01 |
| pH | 8.39 |
| COD (mg/L) | 93.33 ± 18.90 |
| TN (mg/L) | 72.67 ± 15.01 |
