# Supplementary material for: Revealing the functional potential of microbial community of activated sludge for treating tuna processing wastewater through metagenomic analysis
Source: Front Microbiol. 2024 Jul 19;15:1430199. doi: 10.3389/fmicb.2024.1430199 (PMC11294940; doi:10.3389/fmicb.2024.1430199)
Supplement: Supplementary file 6 [file Table_3.DOCX]

**Table S3 Relative abundance of key functional genes and enzymes in fatty acid degradation process.** The enzymes encoded by the second column of genes are shown in the first and last columns, and the third column indicates the relative abundance of functional genes.

| **Enzyme number** | **Gene** | **Relative Abundance (%)** | **Enzyme name** |
| --- | --- | --- | --- |
| 6.2.1.3 | *ACSL* | 0.468426 | long-chain acyl-CoA synthetase |
|  | *ACSBG* | 0.000393 | long-chain-fatty-acid--CoA ligase ACSBG |
| 1.3.3.6 | E1.3.3.6 | 0.00075 | acyl-CoA oxidase |
| 1.3.99.- | *fadE* | 0.043801 | acyl-CoA dehydrogenase |
| 1.3.8.7 | *ACADM* | 0.303209 | acyl-CoA dehydrogenase |
| 1.3.8.8 | *ACADL* | 0.007053 | long-chain-acyl-CoA dehydrogenase |
| 1.3.8.9 | *ACADVL* | 0.001453 | very long chain acyl-CoA dehydrogenase |
| 4.2.1.17 | *paaF* | 0.165458 | enoyl-CoA hydratase |
|  | *fadB* | 0.006247 | 3-hydroxyacyl-CoA dehydrogenase / enoyl-CoA hydratase / 3-hydroxybutyryl-CoA epimerase / enoyl-CoA isomerase |
|  | *fadJ* | 0.054167 | 3-hydroxyacyl-CoA dehydrogenase / enoyl-CoA hydratase / 3-hydroxybutyryl-CoA epimerase |
|  | *ECHS1* | 0.003859 | enoyl-CoA hydratase |
|  | *fadB* | 0.000799 | enoyl-CoA hydratase |
|  | *HADHA* | 0.001944 | enoyl-CoA hydratase / long-chain 3-hydroxyacyl-CoA dehydrogenase |
| 1.1.1.35 | *HADH* | 0.000118 | 3-hydroxyacyl-CoA dehydrogenase |
|  | *fadN* | 0.12582 | 3-hydroxyacyl-CoA dehydrogenase |
|  | *fadB* | 0.006247 | 3-hydroxyacyl-CoA dehydrogenase / enoyl-CoA hydratase / 3-hydroxybutyryl-CoA epimerase / enoyl-CoA isomerase |
|  | *fadJ* | 0.054167 | 3-hydroxyacyl-CoA dehydrogenase / enoyl-CoA hydratase / 3-hydroxybutyryl-CoA epimerase |
| 1.1.1211 | *HADHA* | 0.001944 | enoyl-CoA hydratase / long-chain 3-hydroxyacyl-CoA dehydrogenase |
| 2.3.1.16 | *fadA* | 0.095641 | acetyl-CoA acyltransferase |
|  | *ACAA2* | 0.000659 | acetyl-CoA acyltransferase 2 |
|  | *HADHB* | 0.001091 | acetyl-CoA acyltransferase |
| 1.3.8.1 | *ACADS* | 0.107519 | butyryl-CoA dehydrogenase |
| 1.3.8.5 | *ACADSB* | 0.000258 | short-chain 2-methylacyl-CoA dehydrogenase |
| 2.3.1.9 | *ACAT* | 0.305669 | acetyl-CoA C-acetyltransferase |
| 1.3.8.6 | *GCDH* | 0.050426 | glutaryl-CoA dehydrogenase |
| 6.2.1.20 | *mbtM* | 0.006175 | long-chain-fatty-acid--[acyl-carrier-protein] ligase |
|  | *aas* | 0.052676 | acyl-[acyl-carrier-protein]-phospholipid O-acyltransferase / long-chain-fatty-acid--[acyl-carrier-protein] ligase |
| 5.3.3.8 | *fadB* | 0.006247 | 3-hydroxyacyl-CoA dehydrogenase / enoyl-CoA hydratase / 3-hydroxybutyryl-CoA epimerase / enoyl-CoA isomerase |
|  | *ECI1* | 0.003576 | Delta3-Delta2-enoyl-CoA isomerase |
|  | *ECI2* | 0.008841 | Delta3-Delta2-enoyl-CoA isomerase |
| 5.1.2.3 | *fadJ* | 0.054167 | 3-hydroxyacyl-CoA dehydrogenase / enoyl-CoA hydratase / 3-hydroxybutyryl-CoA epimerase |
|  | *fadB* | 0.006247 | 3-hydroxyacyl-CoA dehydrogenase / enoyl-CoA hydratase / 3-hydroxybutyryl-CoA epimerase / enoyl-CoA isomerase |
| 1.14.15.3 | *alkB1_2* | 0.005053 | alkane 1-monooxygenase |
| 1.18.1.3 | *hcaD* | 0.037233 | 3-phenylpropionate/trans-cinnamate dioxygenase ferredoxin reductase component |
| 1.18.1.1 | *rubB* | 0.029214 | rubredoxin---NAD^+^ reductase |
| 1.1.1.1 | *adhP* | 0.028091 | alcohol dehydrogenase, propanol-preferring |
|  | *yiaY* | 0.018834 | alcohol dehydrogenase |
|  | *E1.1.1.1* | 0.110854 | alcohol dehydrogenase |
|  | *frmA* | 0.060664 | S-(hydroxymethyl)glutathione dehydrogenase / alcohol dehydrogenase |
|  | *adhE* | 0.009866 | acetaldehyde dehydrogenase / alcohol dehydrogenase |
| 1.2.1.3 | *ALDH* | 0.127105 | aldehyde dehydrogenase (NAD+) |
|  | *ALDH7A1* | 0.000264 | aldehyde dehydrogenase family 7 member A1 |
| 1.14.15.3 | *alkB1_2* | 0.005053 | alkane 1-monooxygenase |
